# Histological Features Detected for Separation of the Edible Leaves of *Allium ursinum* L. from the Poisonous Leaves of *Convallaria majalis* L. and *Colchicum autumnale* L.

**DOI:** 10.3390/plants14152377

**Published:** 2025-08-01

**Authors:** Márta M-Hamvas, Angéla Tótik, Csongor Freytag, Attila Gáspár, Amina Nouar, Tamás Garda, Csaba Máthé

**Affiliations:** 1 Plant Cell and Developmental Biology Research Group, Department of Botany, Faculty of Science and Technology, University of Debrecen, Egyetem Tér 1, H4032 Debrecen, Hungary; totikangi@gmail.com (A.T.); freytag.csongor@etk.unideb.hu (C.F.); aminanouar76@gmail.com (A.N.); garda.tamas@science.unideb.hu (T.G.); mathe.csaba@science.unideb.hu (C.M.); 2 Faculty of Pharmacy, University of Debrecen, Nagyerdei Körút 98, H4032 Debrecen, Hungary; 3 One Health Institute, Faculty of Health Sciences, University of Debrecen, Nagyerdei Krt. 98, Theoretical Building, 6th Floor, H4032 Debrecen, Hungary; 4 Department of Inorganic and Analytical Chemistry, Faculty of Science and Technology, University of Debrecen, Egyetem Tér 1, H4032 Debrecen, Hungary; gaspar@science.unideb.hu

**Keywords:** *Allium ursinum*, *Convallaria majalis*, *Colchicum autumnale*, medicinal plants, epidermal peel, epidermal impression, plant identification, poisonous plants, wild garlic

## Abstract

*Allium ursinum* (wild garlic) has long been collected and consumed as food and medicine in the north temperate zone, where its popularity is growing. *Colchicum autumnale* and *Convallaria majalis* contain toxic alkaloids. Their habitats overlap, and without flowers, their vegetative organs are similar. Confusing the leaves of *Colchicum* or *Convallaria* with the leaves of wild garlic has repeatedly led to serious human and animal poisonings. Our goal was to find a histological characteristic that makes the separation of these leaves clear. We compared the anatomy of foliage leaves of these three species grown in the same garden (Debrecen, Hungary, Central Europe). We used a bright-field microscope to characterize the transversal sections of leaves. Cell types of epidermises were compared based on peels and different impressions. We established some significant differences in the histology of leaves. The adaxial peels of *Allium* consist of only “long” cells without stomata, but the abaxial ones show “long”, “short” and “T” cells with wavy cell walls as a peculiarity, and stomata. *Convallaria* and *Colchicum* leaves are amphystomatic, but in the case of *Allium*, they are hypostomatic. These traits were confirmed with herbarium specimens. Our results help to clearly identify these species even in mixed, dried plant material and may be used for diagnostic purposes.

## 1. Introduction

The consumption of wild plants as “medicinal herbs” or food is becoming increasingly popular in Europe, including Hungary. Among edible wild plants, *Allium ursinum* L. has a rapidly growing popularity, due to the propagation of healthy nutrition in the media. It has been harvested with growing popularity in several countries, including Hungary, the Czech Republic, Poland and Germany. It has been a very popular “wild vegetable” in Western and Northern Europe (Great Britain, Scandinavian countries), and in Italy, Serbia, Bosnia and Herzegovina, Ukraine, Russia, the Caucasus, Iran and Turkmenistan for a long time [1,2,3,4,5,6,7,8,9]. As an excellent food, the fresh and boiled leaves are used to add flavor to salads, soups, sauces, savory dishes and salty cakes, such as scones. The bulbs have been used as seasoning for some dishes, such as salads and meats [10,11].

*Allium ursinum* has a wide distribution in the Northern Hemisphere, spreading throughout Europe, Asia Minor, the Caucasus and Siberia to the Kamchatka Peninsula [12]. It has several local names (for example: Ramson, wild or Bear’s garlic, similarly “Bärlauch” in Germany and “Medvehagyma” in Hungary). Its populations inhabit damp but well-drained and shady deciduous forests (for example, in European beech-dominated communities from 1000 to 1750 m a.s.l. in Serbia and in hornbeam, elm and beech forests in Hungary), usually with a high humus content in mountains and lowlands as well [10,13,14,15,16]. In the hilly area of Transylvania, with oak–hornbeam and hornbeam–beech mixture forests, wild garlic can form monospecific stands. In their invasion, not only the optimal circumstances (among others, high nitrogen content and no aluminum in the soil) can be important, but their allelopathic activity as well [12,17]. Wild garlic can be well cultivated as a garden and medicinal plant, and furthermore, there are populations that have been genetically evaluated as "garden escapes” [18].

Consumption of wild garlic is very useful, because the leaves are full of minerals (among others, Se), vitamins and antioxidants, with strong detoxifying properties, and allicin and spirostanol saponins, with antimicrobial and cytotoxic activities [5,8,9,19,20,21]. Special chemicals produced by wild garlic are summarized in excellent review and experimental work articles [1,4,22,23]. The main groups of these components are similar to the beneficial compounds produced by cultivated garlic (*Allium sativum*), but due to some specific components (phytosterols and galactolipid derivatives), it has not only greater positive effects than cultivated garlic on blood pressure and blood chemistry, but unique effects as well [24,25]. In a rat model system, the wild garlic liophylisate-enriched supplementation improved right ventricle systolic function [26], and exerted protection against pulmonary arterial hypertension. The wild garlic liophylisate used was very rich in flavonoids [27]. Due to its sulfuric compounds content (alliin, allicin and others) it has not only a garlic-like scent, but parasite-killing, fungicidal and antibacterial effects, as well. Further actions were found in vivo: prevention of arteriosclerosis, fibrinolytic actions, slowing down the aggregation of blood platelets, anti-cancer and anti-inflammatory effects and strengthening of the human immune system [1,5]. Despite its healing effects, which were written about more than 5000 years ago in Ayurveda and Chinese medicine, and have been proven using in vitro and in vivo model systems and modern methods of molecular biology, analytical chemistry and pharmacognosy, this species is not included in the European and Hungarian official lists of herbal drugs (Ph.Eur.9.0. and Ph. Hg.VIII.) [28,29], so it is used as traditional medicine and dietary supplement [30,31]. It became popular mainly as a culinary, healthy human food [3,10,11,32].

In parallel with the growing popularity of herbs, severe plant poisoning cases have been reported [33,34,35]. The *Allium ursinum* distribution area overlaps with other early spring species having similar habitats, that are about the same height (15–40 cm), and the leaves of which are also simple, slightly elongated and elliptical in shape, with a pointed leaf apex and entire leaf margins. However, many of these species contain special metabolic chemicals that are toxic to humans. Among the species that cause poisoning and have confusingly similar leaves, the most common are *Convallaria majalis* L. and *Colchicum autumnale* L. (Supplementary Table S1) [36,37,38,39,40,41,42].

*Convallaria majalis* L., called the “Lily of the Valley”, is becoming widespread in Europe—living in deciduous forests, up to 1240 m in southern Norway and up to 2300 m in the Alps—partly as a result of their successful reproductive (both vegetative and generative) strategies, and partly because they have been cultivated for ornamental purposes [43]. Their toxicological significance fluctuates from year to year and varies from country to country as well. For example, in Hungary, *Convallaria majalis* was in the top four plant taxa that regularly caused intoxications in the period 2005–2017. It caused 6–20% of all 2464 poisoning cases [44]. In Slovenia, the poisonings with Lily of the Valley occurred in the period 2000–2013 only rarely, and the plant was evaluated as a “mildly poisonous plant” [45]. The human poisonings caused by *Convallaria* frequently concern young children and adults suffering from advanced dementia who chew the berries, stems and leaves, or patients who use it as an herb not professionally, precisely dosed [46,47,48]. *Convallaria* contains many toxins, such as saponins and various others, including around 40 cardiac glycosides in which the main components are convallatoxin (CNT), convalloside and lokunjoside [46,49]. Cardiac glycosides cause digitalis-like toxicity, whereas saponin is responsible for digestive disorders [50]. Various symptoms of CNT poisoning are well described both in animals (especially in cats and dogs) and in humans: salivation, nausea, vomiting, abdominal pain, pupil dilation, slow and irregular heartbeat, hypertension and cardiotonic and blood-clotting actions [51]. Because cardiac glycosides can improve the efficiency of the heart muscle, their use has been known for a very long time [52]. Stansbury et al. [53] still consider *Convallaria,* especially together with *Crataegus,* effective in curing heart disease today. It is important to emphasize that overdose of these extracts or medicines can lead to serious poisonings; these effects were proved by published case reports [52,54].

*Colchicum autumnale* L.—its most common names are “Meadow Saffron” and “Naked Lady”—has a slightly smaller area than the two other species, because its habitats are limited to Ireland, England, Central Europe and North Africa [43,55]. Its populations grow in woodland, clearings, wet meadows, pastures and shady rocky habitats on non-calcareous substrates, up to an altitude of 2.000 m; moreover, it has been cultivated throughout much of the world [55,56]. The first medical use of the bulbs and extracts of *Colchicum* was reported in the first century AD in Unani Tibb, Ayurveda and in *De Materia Medica* by Pedanius Dioscorides, as discussed by work published by Akram et al. [55]. It was mentioned as the most useful and famous agent for removing joint pains, backache and gout, and as a traditional healer of internal injuries [55]. Nowadays, the drug is not recommended for medicinal use due to its poisonous alkaloid colchicine content. The highest concentrations of colchicine are in the seeds and then the corms during the summer, and the amount of colchicine in two or three seeds can be fatal [57]. It is important to highlight that the colchicine content of leaves is not destroyed by heat or boiling and is highly soluble in water. Therefore, it can be rapidly absorbed after oral administration and it is metabolized at body temperature to the more poisonous oxydicolchicine. Given the risks of colchicine, nowadays pharmaceutical preparations of pure isolated colchicine and more often of the less toxic demelkocine are used for acute gouty arthritis, familial Mediterranean fever, and amyloidosis. Unfortunately, all forms of its use carry the risk of overdose and thus often fatal poisonings [58]. Thanks to the relevant properties of colchicine, which binds to tubulin molecules and prevents mitosis of dividing cells, it is a commonly used chemical in chromosomal studies.

Studies on accidental poisonings with plant species are mainly case reports (or their reviews) focusing on the circumstances of poisonings, on the detected symptoms of patients, on the used health care therapy and on the outcome of patients [59,60]. Ng et al. [60] identified the plant species most commonly involved in cases of plant poisoning in Hong Kong (2003–2017) on the basis of morphology and biochemistry to provide clinicians with a reference tool for the diagnosis and management of plant poisoning. Reasons for using the poisonous plants included misidentification (n = 34, 55% of total). In this manuscript, we show the cases of accidental poisonings by the use of *Convallaria majalis* and *Colchicum autumnale* instead of *Allium ursinum* (Supplementary Table S1). These cases highlight why there are so many different opinions on the toxicity and usability of *Convallaria* and *Colchicum*. After all, the toxicity depends on the toxin content of the plant, which is different in plant organs and altered by degree of maturity, differentiation and environmental factors-, as well as on the consumer’s age, body weight, state of health and individual sensitivity, etc. [52,54,59].

**Figure 1:**
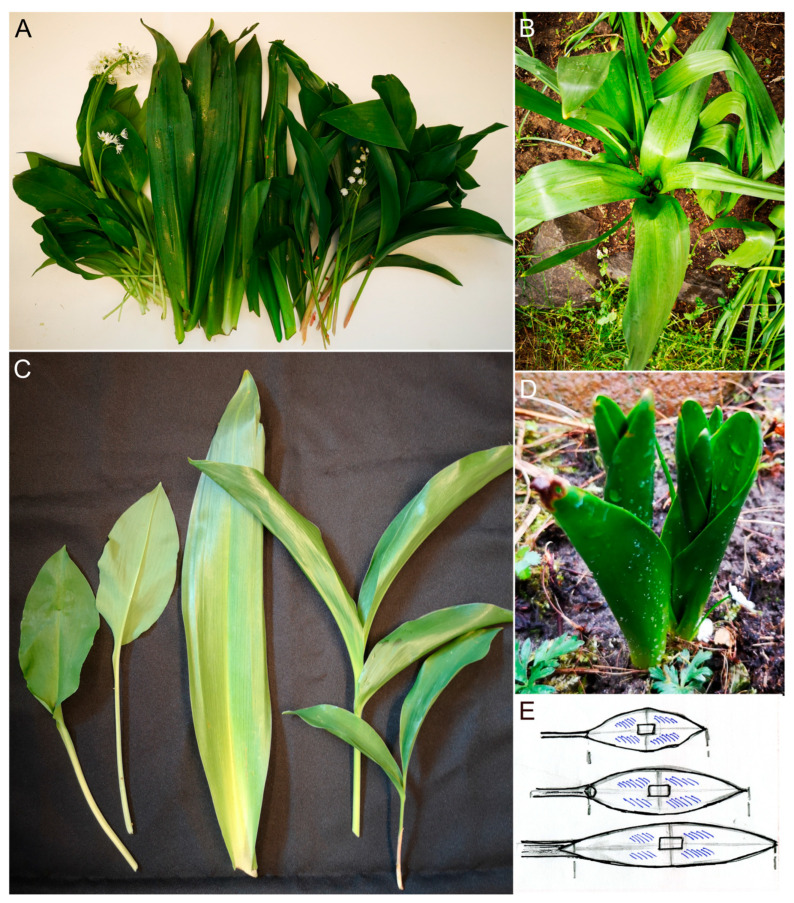
Leaves of the investigated plants. (**A**): Leaves and inflorescences of *Allium ursinum*, *Colchicum autumnale* and *Convallaria majalis*. (**B**): While *Allium* and *Convallaria* produce flowers in the spring, among the *Colchicum* leaves, the ripening capsules are becoming visible. (**C**): Only the leaves of *Allium ursinum* have their own petioles. ((**A**,**C**): From left to right: leaves of *A. ursinum*, *C. autumnale*, *C. majalis*). (**D**): Leaves of *Colchicum autumnale* are the first visible of the three geophytes in spring. (**E**): Drawings show where the sizes of blades were measured and the microscopic preparations (TS—rectangle; peelings—striped areas) were prepared.

Flowering specimens of *Allium ursinum* and *Convallaria majalis* are well distinguishable, but before and after flowering, the leaves of plants can be mixed/confused (Figure 1A, Supplementary Table S1). In spring, *Colchicum autumnale* produces fruits hidden among the leaves instead of flowers (Figure 1B, except *C. autumnale* var. *vernum*). This is the reason why, year after year, during the season of the *A. ursinum* harvest, we can read case reports about poisonings (Supplementary Table S1).

The pharmacological and botanical literature of the three species we examined is very rich, but their focuses are very different. There are review articles that provide accurate botanical, phytochemical and pharmacological overviews of medicinal plants (for example of *A. ursinum* [4] and of *C. autumnale* [55]), but comparative anatomical, and especially histological, analyses of dangerous species that can be confused with edible ones, -such as these medicinal plants we examined-, are still very rare. Mainly, special books of botanical pharmacognosy for microscopic characterization of botanical medicines can help in this type of work [61].

The subjects of our experiments were the populations of these three species that were grown in the same garden (a private garden in Debrecen, Hungary) with a continental climate and sandy soil amended with gardening compost. Leaves of plants were collected and measured, and transversal sections and peels of leaves were investigated using conventional bright-field microscopy (Figure 1).

The main objective of our investigations was to find some easily identifiable morphological/histological, *Allium ursinum*-specific properties, having taxonomic importance to help non-specialists in the safe/secure collection of wild garlic, avoiding misidentification, as well as to help professionals in the essential and accurate identification of consumed plants or drugs in clinical poisonings [58,60]. We have found usable differences in the shape and structure of leaf-blade cross-sections and in epidermis cell types, which were detectable not only on leaf samples from 2022 and 2023, but on specimens in Herbarium Univ. Debreceniensis, collected in different years and regions of Europe. Our results can be useful not only for humans but for veterinary medical diagnostic laboratories as well [62].

## 2. Results

### 2.1. The Anatomical Characters of Leaves

Among the three geophytic plants, *Colchicum* leaves emerged first in early April (Figure 1D), followed by *Allium* in mid-April and *Convallaria* leaves only at the end of April. This order is typical in the garden every year. In May the *Colchicum* specimens have 3–4 leaves, of which the outer two are well developed, having the significantly largest lamina-length parameters (Figure 1A–C and Table 1). Although the *Convallaria* leaves started to grow later, by May they reached a significantly greater blade length than the *Allium* ones (Figure 1A,C and Table 1). In May of 2023 we measured the length of petioles as well, and *Convallaria* had the higher average value (*Convallaria*: 9.64 ± 2.5, *Allium*: 7.26 ± 3.1, mean ± StD in cm, having a significant difference in paired *t*-test, *p* = 0.024). Unlike the blade length, the blade-width values showed no remarkable differences between the three species (Table 1).

**Figure 2:**
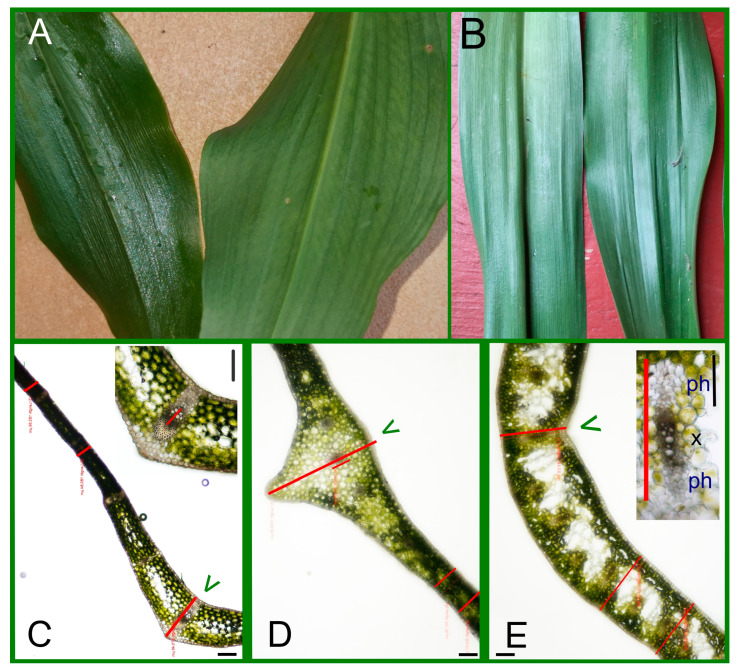
The lower surfaces of leaf blades with the midveins and the measured histological parameters of leaf blades. (**A**): Abaxial (lower) surfaces of *Convallaria majalis* and *Allium ursinum* leaves; (**B**): from right to left, the adaxial (upper) and abaxial surfaces of leaf blades of *Colchicum autumnale*. Measurement points (red lines) in the TSs of leaf blades of *Convallaria* (**C**), of *Allium* (**D**) and of *Colchicum* (**E**). (**E**): The inserted figure shows the higher magnification image of the bicollateral midvein in a *Colchicum* leaf. The green arrowheads show the adaxial surfaces of leaf blades at the midveins (hand-made native sections). The red lines show the measured parts of the preparations. Leaf cross-sections at the midvein of the three species examined differ in shape. Bars: 200 μm.

**Figure 3:**
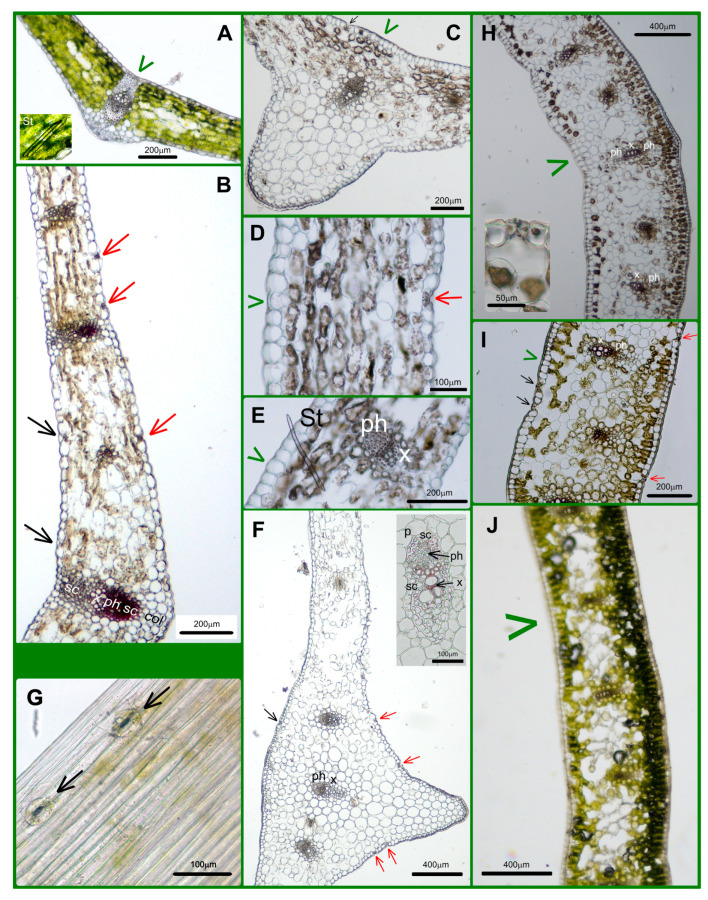
The transversal sections of leaf blades. (**A**,**B**): *Convallaria majalis*, (**C**–**G**): *Allium ursinum*, (**H**–**J**): *Colchicum autumnale.* The green arrowheads show the adaxial (upper) surfaces of leaf blades. The presence of stomatal complexes is indicated by black arrows on the upper and by red arrows on the lower surfaces. (**G**): Stomatal complexes (black arrows) above the midvein on the adaxial surface of an *A. ursinum* leaf. (**H**): The inserted image shows the stomatal complex. (St.: styloid Ca oxalate crystal, x: xylem, ph: phloem, sc: sclerenchyma, col: collenchyma, p: parenchymatic sheath). (**A**,**J**): Hand-made sections, (**B**–**F**,**H**,**I**): microtome-sectioned preparations (**B**) and the inserted image of the midvein in (**F**) are stained with Phloroglucinol–HCl; the others are unstained.

We made the transversal sections (TSs) in the middle part of blades containing the midvein and, parallelly, some smaller ones (Figure 1E and Figure 2A–E). In this part of the *Allium* leaf blade, the zone of the midvein was very prominent (Figure 2A,D and Table 1). The diameter of the central vein itself was not large (250–290 μm, Table 1), but the strongly prominent central zone of blade contained not only the midvein but, parallelly, two smaller ones as well. These veins were embedded in 20–25 layers of parenchymatic cells with decreasing chloroplast content towards the lower side (Figure 2D and Figure 3F). The middle part of the leaf blade of the two other species (*Convallaria* and *Colchicum*) contained only a single midvein (Figure 2C,E and Figure 3A,B,H).

There was no connection between the leaf thickness at the midvein and the diameter of the midvein measured in the TSs (Table 1). The midvein of *Convallaria* was supported not only by sclerenchyma fibers but, beneath the lower epidermis, by a collenchyma girder as well (Figure 2C and Figure 3A,B and Supplementary Figure S1). The middle part of the *Colchicum* leaf showed threading instead of protrusion (Figure 2E and Figure 3H) and contained the biggest of three, a bicollateral midveins without any supporting tissues (Figure 2E). The other longitudinal veins were collateral (Figure 3H–J). The average thickness of leaves showed significant differences between the three species as well (Table 1). *Colchicum* leaves were the thickest and *Convallaria* leaves the thinnest (Figure 2C–E, Table 1).

The anatomy of leaves in TSs showed the isolateral structure of Monocotyledon species with some small, but interesting, differences, which are summarized in the Discussion Section. Styloids are characteristic of Monocots, showing large, much elongated prisms of calcium oxalate crystals having oblique or chisel-shaped ends. They were detected in *Convallaria* and *Allium* leaves (Figure 3A,E).

### 2.2. Differences in Epidermal Peels

**Figure 4:**
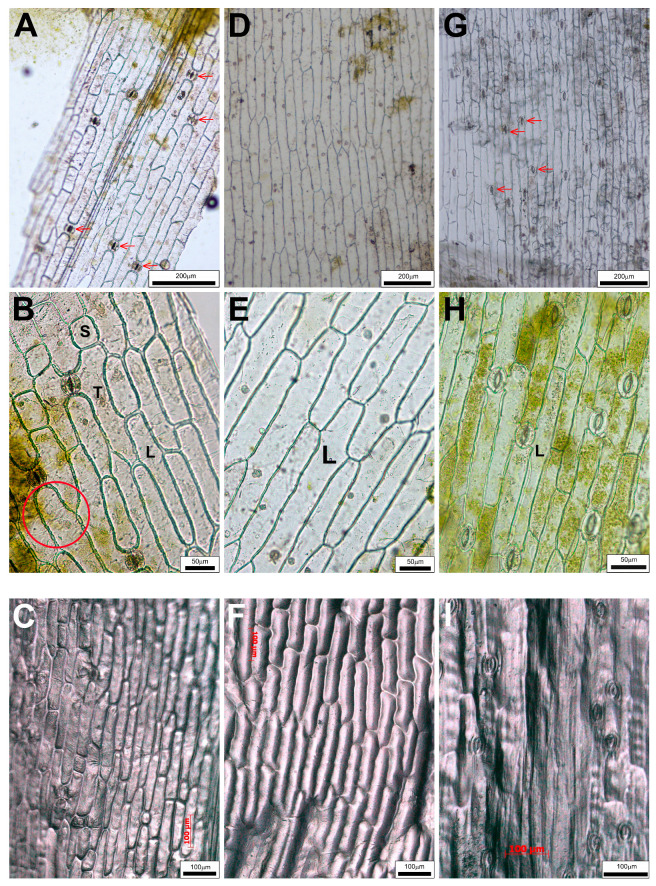
Adaxial surfaces of leaves. (**A**–**C**): *Convallaria majalis*, (**D**–**F**): *Allium ursinum*, (**G**–**I**): *Colchicum autumnale.* (**A**,**B**,**D**,**E**,**G**,**H**): Unstained peelings, (**C**,**F**,**I**): Polymer (PDMS) imprints. (**A**–**C**): Epidermal pavement cells can be differentiated into “long” (L), “short” (S) and “T” cells (T) based on their shape in the adaxial surface of *Convallaria*. (**D**–**I**): Adaxial epidermal layers of *Allium* and *Colchicum* consist of only the “long” (L) type of pavement cells. (**A**,**G**): Red arrows indicate the stomata. (**B**): The cell wall pits are clearly visible in the area marked with a red circle.

**Figure 5:**
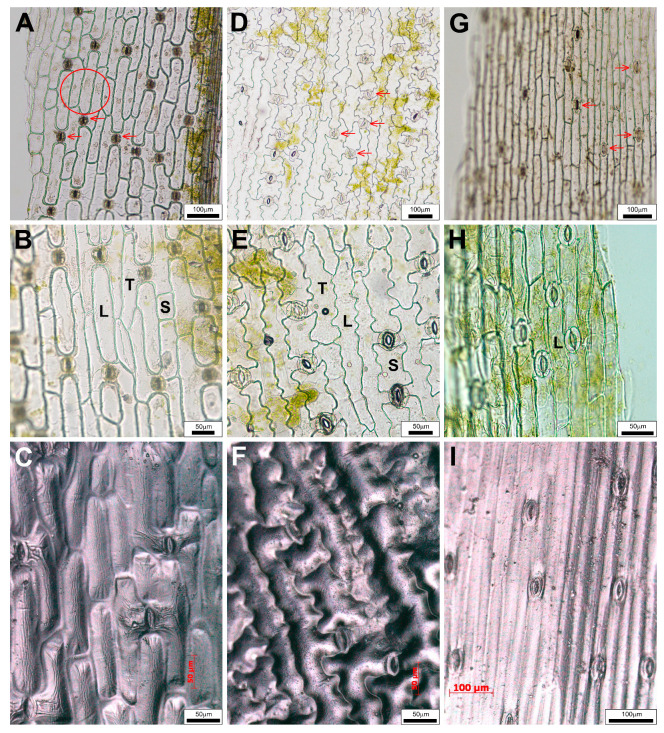
Abaxial surfaces of leaves. (**A**–**C**): *Convallaria majalis*, (**D**–**F**): *Allium ursinum*, (**G**–**I**): *Colchicum autumnale.* (**A**,**B**,**D**,**E**,**G**,**H**): Unstained peelings, (**C**,**F**,**I**): polymer (PDMS) imprints. ((**A**–**F**): Epidermal pavement cells can be differentiated into “long” (L), “short” (S) and “T” cells (T) based on their shape. (**A**,**D**,**G**): Red arrows indicate the stomata. (**A**): The cell wall pits are clearly visible in the area marked with a red circle. (**D**–**F**): Only the *Allium*’s abaxial pavement cells have wavy cell walls).

Peelings of *Convallaria* blades showed that pavement cells are elongated in the direction of the longitudinal axis of the leaf, the cell wall is thickened and simple pits are visible. They are in three different shapes. The name “long cell” (L) was given for the most abundant epidermis cells of oblong blades (Figure 4A,B). Among these longish cells, there were some shorter ones (“S” in Figure 4B). The long cells adjacent to guard cells of stomatal complexes are slightly elongated towards the stomata, giving them a “T” shape (Figure 4B). These types of cells were detectable not only in both adaxial and abaxial epidermal layers of *Convallaria* (Figure 4A,B and Figure 5A,B), but in the abaxial epidermis of *Allium* as well (Figure 5D,E). These cell types were not characteristic for adaxial peels of *Allium* (Figure 4D,E) and for both surfaces of blades of *Colchicum* (Figure 4G,H and Figure 5G,H).

We measured the area of these cells, and the statistical comparison of data confirmed the differences between these cell types (Table 2).

In *Colchicum*, neither the cell shapes nor the statistical comparison of the area values justified the existence of two (long and T) cell types (Figure 4G–I and Figure 5G–I). A very important feature is that only *Allium*’s abaxial pavement cell walls are wavy, while those of all other pavement cell types are straight (Figure 4 and Figure 5). The main explanation for the fact that the upper epidermis of *Allium* consists only of long cells is the absence of stomatal complexes (Figure 4D–F and Table 3). The data in Table 3 proved that while both *Convallaria* and *Colchicum* leaves are amphystomatic, *Allium* can be called hypostomatic.

While we did not see stomata on the peeled wild garlic upper leaf blades (Figure 4D,E), the cross-sections show some above the midvein (Figure 3C,F, black arrows). The presence of these stomata among the typical narrow, elongated pavement cells covering the midvein zone was proved with extra peels (Figure 3G). Interestingly, the *Colchicum*’s longest leaves were not covered with the largest pavement cells, but the smallest *Allium* leaves were (Table 2). The stomata frequencies of the two amphystomatic species showed an opposed tendency; in the case of *Colchicum*, not the lower, but the upper epidermis contained more stomata (Table 3). The Amaryllis-type stomatal complexes were anomocytic, without subsidiary cells (Figure 4 and Figure 5).

### 2.3. Differences Between the Parameters in 2022 and 2023

In a comparison of the same characters of the same populations in 2022 and 2023, similar tendencies with some small differences were detected. In 2023, the first- and last-appearing *Colchicum* and *Convallaria* leaves’ average lengths were smaller than in 2022. The areas of upper epidermis cells of *Convallaria* and *Colchicum* were significantly bigger in 2023 than in 2022, even though the leaves were smaller in size and a bit thicker (Table 1 and Table 2). The “long” cells were most frequently cells of the lower epidermis of *Convallaria,* for which the average size was increased significantly in 2023, and, in parallel, the number of stomata among pavement cells was slightly decreased, but not significantly (Table 2 and Table 3). *Allium* data were similar in both years—except for the average thickness of lamina measured at midvein and the higher number of stomata, which may indicate more optimal conditions. These results do not affect our previous findings (Table 1, Table 2 and Table 3).

### 2.4. Impressions of Leaf Surfaces

It is often difficult to separate the epidermis from the hypodermal tissues, for example, in the case of the upper epidermis layers of *Convallaria* and *Colchicum*. This was the first reason that we tried other methods as well. The other reason was to confirm or reject the differences detected in our studied populations by the results of the herbarium specimens collected in very different areas and times. Impressions of a special polymer (Figure 4C,F,I and Figure 5C,F,I) and simple nail polish (Supplementary Figure S2) were as nice and rich in detail as the ordinary epidermal peelings. The impressions of pressed and flattened leaf surfaces of herbarium specimens gave less detailed pictures (Supplementary Figures S3–S5), but they still proved that *Allium* can be separated from the other two species on the basis of the epidermis. Either the absence of stomata among the elongated cells (upper surface character) or cells with wavy cell walls around the stomatal complexes (lower epidermis) are distinguishing features. These histological characters that can be easily examined may contribute to the clear identification of fresh leaves and dried drugs of *A. ursinum*.

## 3. Discussion

The finding that the leaves of *Allium ursinum* are narrower, softer and duller than those of *Convallaria majalis* often does not provide sufficient help for their separation (Figure 1 and Figure 2A). The leaves of *Colchicum autumnale* are particularly shiny and can be much longer than those of *Allium* (Figure 1 and Figure 2A,B); despite this, there are mistakes that cause serious poisonings (summarized in Supplementary Table S1). Our leaf measurement data proved both similarities in the widths of blades and significant differences in the lengths and thicknesses of blades between the three investigated species (Table 1). Important advice for harvesting is to collect the *Allium* leaves together with their peduncles individually. This is something that is not fulfilled in the case of *Convallaria* and *Colchicum*, since their leaves grow in twos and threes around each other (Figure 1). The garlic smell of the leaves is a great mark, and the prominent midvein and sparser venation also help in the recognition of wild garlic. In connection with cases of poisonings, it is important to note that the elderly, with a weaker sense of smell, may not recognize the difference between the plants [58].

It is well known that the subspecies, ecotypes of *Allium ursinum,* can show significant differences in the length and width of leaf blades, the length of petioles, the dry mass of leaves and their content of biologically active components [1]. Three ecotypes of wild garlic were investigated in a botanical garden (Lublin, Poland) during 2007–2009. The leaves were grown from April until May, and data on the length and width of blades and the length of petioles varied in the ranges of 70–362, 22–66 and 25–144 mm, respectively [1]. Our data fit into these wide intervals, but they are mostly similar to the April of 2007 data, when not only the vegetative organs but also the flowers were smaller. In Debrecen, Hungary (47.515° N, 21.643° E), the climate and soil may be less damp and optimal for these species than in Lublin, Poland (51.23° N, 22.56° E). What strengthens our conclusions is that the data of our studied population proved to be less variable.

In the case of *Convallaria*, the dense parallel veins are well visible on fresh leaves and as traces in peelings and impressions of epidermal layers as well (Figure 2A,B, Figure 4 and Figure 5 and Supplementary Figure S3). *Allium* leaves are softer and duller than *Convallaria* leaves, but *Colchicum* leaves are the dullest and have the highest average thickness parameters of blades (Table 1). The structures of mesophylls are similar, typical of Monocotyledon species. They have uniserate upper and lower epidermises covered by distinct cuticles. Guard cells of stomata are not sunken, and the *Allium* and *Convallaria* leaves do not have distinct palisade and spongy mesophylls but have parenchyma cells of a rather uniform size and shape (Figure 2C,D and Figure 3A–F). On the contrary in *Colchicum* leaves below both epidermal layers there are palisade parenchymatic cells (Figure 3H–J). On difficult-to-separate peelings, the palisade parenchymatic cells are visible as tightly fitting circles. The palisade ratio, the average number of palisade cells covered by one pavement cell (a rarely used microscopic character in Monocotyledon plants because of the more common homogenous structure [54]), varied between 4 and 9 (Supplementary Figure S6).

On the basis of TSs, peelings and impressions *Convallaria majalis* have the typical unifacial, amphystomatic leaf (Figure 2C, Figure 3A,B, Figure 4A–C and Figure 5A–C, Supplementary Figures S2A,B and S3). Both surfaces of leaves are covered by the same cell types (“long”, “short” and, next to stomata, the “T” cells in shape). As usual, the stomatal density is higher on the lower epidermis (Table 3). Mesophyll cells are horizontally elongated giving a compact tissue with intercellular air spaces with a maximum diameter of 10 μm. The number of cell layers is 6–7, and in the central layer, one series of veins occurs with xylem facing towards the upper epidermis. Veins are supported by sclerenchyma girders, which are fixed about every second vein to the epidermal layers. The midvein is covered by a parenchymatic sheath as well, and supported by collenchyma cells, giving the protrusion of the midvein in the lower surface of the blade (Figure 3A,B and Supplementary Figure S1). Over time, the central parenchymatic cell layers (1–3) lose their chloroplast content—especially next to the central vein (Figure 2C and Figure 3A).

*Allium ursinum* has a unifacial leaf structure containing homogeneous mesophyll with a little asymmetry (Figure 2D, Figure 3C–F, Figure 4D–F and Figure 5D–F). Below the adaxial epidermis without stomata, two layers of parenchymatic cells rich in chloroplasts result in a compact tissue with only 25–40 μm intercellular spaces. It is not easy to identify the subepidermal laticifer cells with taxonomic importance [63]; only the lack of chloroplasts helps to separate them from photosynthesizing mesophyll cells (Supplementary Figure S7). In the direction of the lower epidermis, the mesophyll is composed of 5–6 layers of typical spongy cells with 70–100 μm of intercellular spaces. One series of vascular bundles with a sheath of parenchyma and/or sclerenchyma fibers, but without sclerenchyma ridges, is embedded in the central part of the mesophyll (Figure 3F). Interestingly, in veins, the phloem elements are oriented adaxially! Our observations confirm the data of Mashayekhi and Columbus [63], that the TSs of flattened leaves of *Allium ursinum* do not contain palisade mesophyll, and the distribution and orientation of vascular bundles are characterized as “single row and inversely oriented”. *A. ursinum* is divergent from all other Old World *Amerallium* (subgenus of *Allium*) species based on leaf morphology and anatomy, according to Arber (1961), published in [63]. On the basis of our preparations, the lower surfaces of leaves are covered by cells having wavy cell walls and three types of cell shape. Based on the presence of Amaryllis-type anomocytic stomatal complexes on the abaxial surfaces, *A. ursinum* leaves are hypostomatic (Figure 4D–F and Figure 5D–F and Supplementary Figures S2C,D and S4).

The unifacial, amphystomatic leaf blade of *Colchicum* is covered only by elongated cells of the same shape (uniform upper and lower epidermises, Figure 4G–I and Figure 5G–I and Supplementary Figures S2E,F and S5). However, TSs show a heterogenous mesophyll containing palisade and spongy parenchyma as well (Figure 2E and Figure 3H–J). The palisade mesophyll with a high chloroplast content is 1–3 layers beneath the abaxial epidermis and only 1 layer beneath the adaxial one (Figure 3J). It is interesting that on this “isobilateral” blade, the stomatal density of the upper epidermis is higher than that of the lower one (Table 3). We suppose that this inverted structure, compared to the general one, is due to the longer exposure of the abaxial surface to light being rolled around the stem. The central spongy mesophyll (about 10–12 layers of cells) contains one series of veins, the bicollateral midvein and the smaller collateral ones (Figure 2E and Figure 3H–J), of which about every fourth remained in connection with the epidermis by mesophyll cells, because in differentiated leaves the central parenchymatic cells differentiated to big intercellular/mucilage cavities (Figure 3J). Surprisingly, in the TSs, the distances between two parallel veins were the smallest in *Colchicum* (280–540 μm), smaller than in *Convallaria* (530–750 μm), and the biggest in *Allium* (1500–2000 μm)! Vein traces are visible in some peelings of *Colchicum* (Supplementary Figure S6), but not on *Allium* ones. We think that the visibility of these veins depends on the presence or absence of the vessel–epidermis connection and the thickness of the blade.

Based on our preparations made in 2022 and 2023 and our measured data, these basic histological properties can be considered stable (Table 1, Table 2 and Table 3). Only the weather could cause some differences between years. The earliest developing *Colchicum* seems to be the most sensitive to this. *Colchicum* leaves were shorter, but wider and thicker in the wetter 2023 year. At the same time, we did not examine the capsules or seed production, so we have no data on the ratio of the “cost” of generative and vegetative organs. In early spring, this plant also uses energy for the ripening of seeds and for the development of leaves. The period of photosynthesis is short, from the end of March until the middle of July. After the seeds are scattered, the leaves also die. In 2021 March, April and May the average daily temperatures were lower (daily averages: 5.82, 8.79 and 14.6 °C, with −8, −4.2 and 2 °C daily minimum temperatures) than in 2022 (5.2, 9.5, 17.8 °C, with −1.2, 2.6 and 4.1) and in 2023 (7.5, 10.4, 17.4, with −2.4, −0.9, 5.8 daily minimum temperatures) registered by HungaroMet [64]. The summarized precipitation of these 3 months was similar in 2021 and in 2022 and higher in 2023 (2021: 72.9; 2022: 77.6 mm and 2023: 151.4 mm). An interesting question is which period’s values are decisive for these geophyte plants? During this short period, *Colchicum* can produce the reserve nutrients by photosynthesis and accumulate them in a new corm. ATP from stored materials enables autumn flowering, seed and fruit formation, which ends only in the spring of the next year, in the time of the new leaves’ differentiation. How much energy do the new leaves spend on growth, and how much on new storage? It is proven that the development of reproductive organs and secretion of nectar have a significant cost for the plants, and these metabolic processes, for example, the nectar production of *Allium ursinum*, are moreover highly sensitive to various ecological factors [16,65]. It is known that despite the high energy cost, the environment does not greatly affect the concentrations of bioactive compounds that are essential for the plant. Burton et al. [21] compared the allicin concentration and its antibacterial properties against *Bacillus subtilis* of different wild garlic populations growing in different sites (natural habitats and roadsides). They found that the leaf with the biggest area did not have the greatest activity, but the smaller ones did. Green leaves were substantially more active than brown leaves. The explanation was that the plants being grown in suboptimal conditions, and even more so the plants being stressed, produced smaller leaves, but protective chemicals in higher amounts. Our samples were not chemically analyzed. But if the environment was suboptimal for *Allium*, it was similar for the two other species as well. Herden et al. [18] used molecular genetic analyses of nuclear ITS and ETS sequences and plastidic *trn* L-*rpl* 32 and *trn* L-*trn* F spacer regions to compare the genetics of eleven *Allium ursinum* populations from Germany, and no variation was detected within the species. Moreover, sequences of populations from Belfast, Ireland, did not differ from populations from Germany. The theoretically low genetic variability with the relatively similar environmental parameters in 2021–2023 can explain the stability of the measured anatomical parameters of our wild garlic samples (Table 1, Table 2 and Table 3). In contrast to *Colchicum*, the lifespan of *Convallaria* and *Allium* leaves, and the season of photosynthesis, is longer than the period of reproduction, so -they survive the flowering and, thereafter, the ripening of fruits and seeds-, ensuring a more stable food storage for the next year’s leaf differentiation. Jandl et al. [66] published work indicating that the active growth period of *A. ursinum* lasts for 3.5–4 months, starting in early spring between late February and early March, and ending before the full development of tree leaves, in the northern Vienna Woods. A high assimilation rate provides rapid development and the accumulation of nutrients stored in the bulbs [66]. Leaves are rich in biogenic minerals [20], but they can accumulate potential toxic minerals as well, not only from soil, but from the atmosphere as well [8]. The above-ground parts abruptly wither as summer arrives [66]. *Convallaria* leaves could be visible even until the beginning of the cold period. In our study, the wetter spring of 2023 primarily caused a significant increase in the thickness of the early-appearing *Colchicum* and *Allium* leaves. Leaves were more turgid, but not longer. In case of *Allium*, an elevated number of stomata was detected. The size of epidermal pavement cells showed slightly higher values in 2023, accompanied by an increase in leaf width only in the cases of *Colchicum* and *Convallaria*. But the relative proportions of the measured anatomical data of the three species did not change in the two years examined.

These three species are cosmopolitan in the Northern Hemisphere. Climate change may cause them to appear in previously unfavorable areas, and of course, they may disappear as their habitat dries up. Furthermore, they are all popular as imported foreign garden and indoor plants even in countries without their natural habitats. Global trade in plants can increase the number and types of plants that can cause poisonings. Case reports of poisonings show the importance of identification of plant species responsible for toxicosis to establish an accurate diagnosis and to plan the appropriate intensive health care [33,67,68,69,70]. We believe that the anatomical differences of leaves of the three investigated medicinal plants we have identified and shown in this article can be verified from leaf remnants in both wet and dry conditions, making them suitable for establishing a preliminary taxonomic hypothesis when a diagnosis of intoxication is made. We believe that the histological database of plants should also be expanded. If the operator (a doctor or a laboratory technician) uses plant anatomy and histology examinations and knowledge and is able to identify one or more candidate species, and these plant species have already been characterized with molecular approaches, a rapid screening technology based on target PCR can be used for an accurate, confirmatory identification. In the researchers’ opinion, when primers and/or probes have already been developed, the TaqMan or SCARs techniques are the most suitable molecular techniques for plant identification [69].

Nowadays, progress in the field of DNA-based methods for accurate plant identification, including herbs—even in mixed samples—is being made. This is helped by increasing sequence coverage and the populating of dedicated public databases, better quality of the genetic analyzers, new generations of devices, more and more alternatives in Real-Time PCR, possibilities in bioinformatics and, in parallel with these, decreasing analytical times and processing costs [69]. However, accurate plant genetic databases must contain sequences from reliable sources. The reliable sources of DNA are plant specimens determined by classical methods, based on taxonomically useful anatomical features. Therefore, there is still a need for herbariums (classical collections of field-collected and identified plant specimens) and specialists skilled in plant taxonomy and plant anatomy.

## 4. Materials and Methods

### 4.1. Plant Materials

For the morphological and histological investigation of the three species, the specimens were collected in the same garden in Debrecen (Hungary, 47.515° N, 21.643° E), on 13 May 2022. and on 15–26 May 2023. The plants were growing in semi-shaded parts of the garden for more than 15 years, producing flowers and seeds. Their original habitats were as follows: *Allium ursinum* L.—Bihar/Bihor Mountains (Romania), *Colchicum autumnale* L.—uncultivated garden in Mátra Mountains (Hungary), *Convallaria majalis* L.—unknown. This plant is the characteristic species of the woody plant community called “*Convallario-Quercetum roboris*”, growing on the sandy soil of the Great Hungarian Plain, so around Debrecen as well. It is very common as an ornamental plant in Hungarian gardens. In our garden, plants live in separate, small (20–50 pc), slowly growing populations. Thanks to the common habitat (soil and climate), the anatomical differences can only be attributed to the different genetic background—they are not influenced by different environmental factors.

### 4.2. Anatomical and Histological Investigations of Leaves

During our investigations, we focused on foliage leaves, but for identification, of course, other organs of the plants were investigated as well. Leaves of 10–15 different plants of the populations were measured (Figure 1A,C). The tiny leaves of *Allium* seedlings were not collected, because it is known that the seedling develops only one foliage leaf in the first year, and it has a rather small area [14]. The more than one-year-old bulbs produce multiple leaves. We measured the length and the maximum width of the leaf blades. Transversal sections at the middle part (Figure 1E) of 10 fresh leaves (having extents around the average) were made by hand and examined with a bright-field OLYMPUS DP73 microscope and measured by OLYMPUS Cell-Sens-Dimension Software. Three preparations per leaf were measured at the midvein and at 2-2 further points, so far from the midvein, where the leaf thickness is relatively constant (Figure 2C–E). We measured the diameter of the midvein as well. For statistical analysis, the average values of 3 preparations per leaf were used as one data point (Figure 2C–E).

For better quality pictures, TSs were made by microtome as well. Samples of plant organs were fixed and stored in WEG (water/ethanol/glycerol; 1/1/1; *v*/*v*/*v*) for some weeks and before cryosectioning the samples were transposed into 40% (*w*/*v*) sucrose (Reanal, Budapest, Hungary) dissolved in PBS for dehydration (2 × 15′ vacuum, then in a new solution overnight). Ten-to-fifteen μm thick sections were made by cryosectioning with a Leica Histoslide 2000 microtome as we previously described [71,72]. Preparations were investigated without staining or were stained with Phloroglucinol–HCl solution to highlight the lignified cell walls of sclerenchyma fibers and vessel elements [61,73,74].

### 4.3. Epidermal Peels and Impressions

Epidermal peels of fresh leaves were prepared around the central part of the leaves (Figure 1E, dashed parts of leaves). Polymer and nail polish impressions/imprints of fresh and dried leaves were made as well.

For special polymer imprints, a 10:1 mixture of polydimethylsiloxane (PDMS) oligomer and cross-linking agent (Sylgard 184, Dow Corning, Midland, MI, USA) was used [75]. A thin layer (about 3 mm) of PDMS was gently poured onto the clean surfaces of fresh leaves and was left at room temperature for a night to slowly reticulate. After complete solidifying, the PDMS pieces were peeled off from the leaves and investigated using an inverted microscope (Axio Observer A1, Zeiss, Oberkochen, Germany) equipped with a CCD camera.

Clear nail polish was painted on both the upper and lower surfaces of leaves. After drying, the nail polish layer was removed with fine forceps [74]. Nail polish impressions embedded in 50% glycerol are usable similarly as peelings. This method is useful for the investigation of not only fresh leaves but especially that of dry/dried material, such as drugs and herbal specimens, whose leaves are not allowed to be damaged by making peels. The investigated herbal specimens were from Herbarium Univ. Debreceniensis/Herbarium of Debrecen University [76]: (1). *Convallaria majalis* L. collected in Tokaj, Garden of Károly Almássy, Hungary, around 48.123° N 21.409° E, 18 April 1948. (2). *Convallaria majalis* L. Keszthelyi hg. Csókakő, Hungary, 46.8174° N 17.2389° E, 25 April 2017. (3). *Allium ursinum* L. Keszthelyi hg., Rezi-vár, West Hungary, 46.8595° N, 17.2346° E, 30 March 2017. (4). *Allium ursinum subs. ucrainicum*, Sărata Monteoru 45.1026° N, 26.3826° E, Southeast Romania, alt. circa 396 m, 2014. (5). *Colchicum autumnale* L. Postojna, Slovenia, 45.77919° N, 14.20671° E, 3 May 2013. (6). *Colchicum autumnale* L. Kömörő, Szatmár region of Hungary, around 48° N, 22° E, 19 May 1946.

Peels and nail-polish imprints were investigated with the OLYMPUS DP73 microscope.

ImageJ 1.53t software was used for the evaluation of peels and for the area of epidermis cell measurements.

### 4.4. Statistical Analysis

Comparison of data, calculation of averages and standard deviations and evaluation of the significance level of differences between data were done with the aid of Sigma Plot^®^ 11.0 software by using One-way ANOVA statistical analysis.

## 5. Conclusions

*Allium ursinum*—producing the same healing components as garlic, but in different concentrations and with some special chemicals—has been used for thousands of years in the Northern Hemisphere. Despite the increasing cultivation, harvesting of wild garlic leaves in natural habitats—except in countries where they are protected—is typical, which carries the risk of mixing them with leaves of toxic species, mainly of *Convallaria majalis* and *Colchicum autumnale*. During harvesting of medicinal plants, the assessments—not only those of herbalists—are very important. The fact that all three species were grown in the same garden allowed us to collect samples for a comparative anatomical analysis in the spring seasons of 2022 and 2023. *Colchicum autumnale* had the significantly longest, widest and thickest laminae. TSs showed unique characteristics for all three species. We distinguished different cell types in the epidermis and indicated “long-”, “short-” and “T-” types of pavement cells. We measured the area of these cells, and the statistical comparison of these data confirmed the differences between these cell types, of which the “long cells” of *Allium* were the largest. The most important features presented are that wild garlic leaves are hypostomatic and covered by very different upper and lower epidermal layers. The amphystomatic leaves of *Convallaria* and *Colchicum* are covered by similar upper and lower epidermal layers consisting of three and one type(s) of pavement cells, respectively. A straight cell wall was the general feature. Having wavy cell walls was the peculiarity of the abaxial epidermis of *Allium*. Thus, peels or imprintings of wild garlic are distinguishable from those of the other two species even in mixed, dried plant material. In our opinion, the results of our comparative histological investigations, proven with data of two years and from herbarium specimens, can help to clearly identify these species. Histological identification may be used in the diagnosis of accidental intoxications. We can contribute our results to various databases and information networks under development to distinguish plants that can be confused.

## Figures and Tables

**Table 1:** The morphological and anatomical characters of the three species in May of 2022 and 2023.

Year		*Convallaria majalis*	*Allium ursinum*	*Colchicum autumnale*	*p*
average leaf lamina length (cm)	2022	18.55 ± 3.33 ^b^	13.30 ± 2.07 ^c^	**38.10 ± 3.52 ^a^**	<0.001
	2023	16.37 ± 3.44 ^b^	13.46 ± 3.98 ^c^	**29.19 ± 3.64 ^a^**	<0.05
	2022 ↔ 2023	* *p* = 0.026	NS	* *p* = 0.001	
average of the max. leaf widths (cm)	2022	5.40 ± 1.06	4.836 ± 1.16	**5.370 ± 1.347**	NS
	2023	5.63 ± 1.43 ^a^	4.39 ± 1.68 ^b^	**6.04 ± 0.78 ^a^**	<0.05
	2022 ↔ 2023	NS	NS	* *p* = 0.037	
the thickness of leaves at midveins (µm)	2022	480.610 ± 61.77 ^c^	**1330.17 ± 234.08 ^a^**	742.880 ± 92.36 ^b^	<0.005
	2023	444.20 ± 80.40 ^c^	**1813.50 ± 336.40 ^a^**	839.91 ± 76.15 ^b^	<0.05
	2022 ↔ 2023	NS	* *p* = 0.002	* *p* = 0.020	
average thickness of leaves (µm)	2022	190.32 ± 19.89 ^c^	429.40 ± 46.55 ^b^	**726.73 ± 123.27 ^a^**	<0.005
	2023	195.90 ± 17.99 ^c^	447.90 ± 50.44 ^b^	**803.07 ± 93.16 ^a^**	<0.05
	2022 ↔ 2023	NS	NS	NS	
diameter of midvein (µm)	2022	145.30 ± 18.88 ^c^	291.2 ± 50.6 ^b^	**400.30 ± 62.0 ^a^**	<0.001
	2023	152.00 ± 34.89 ^c^	287.60 ± 46.42 ^b^	**460.69 ± 72.91 ^a^**	<0.05
	2022 ↔ 2023	NS	NS	NS	

Mean ± SD, n: 10, ^a,b,c^: indicate the significantly different groups of the same year and * the significant differences between the same parameters in 2022 and in 2023 (NS, not significant, because *p* > 0.05). (One-way ANOVA, analysis of variance, on ranks, then multiple comparison by Dunn’s or Tukey’s method.) The biggest values of categories are highlighted in bold.

**Table 2:** Epidermis cell types and their areas in 2022 and 2023.

Area µm^2^ Year		*Convallaria majalis*			*Allium ursinum*			*Colchicum autumnale*
Cell Types		Short	T	Long	Short	T	Long	Long
upper epider-mis	2022	1763.3 ± 490 ^c^	5755.9 ± 1401 ^b,d^	6347.2 ± 1693 ^b^	-	-	**12** **,** **489.5 ± 2705 ** ^a^	7221.9 ± 1635 ^b,d^
	2023	2658.1 ± 784 ^c^	7081.6 ± 1028 ^b,c^	8238.6 ± 1465 ^a,b^	-	-	**11** **,** **040.9 ± 2534 ** ^a^	9859.2 ± 3588 ^a,b^
	2022 ↔ 2023	* *p* = 0.003	* *p* = 0.015	* *p* = 0.01			NS	* *p* = 0.01
lower epider-mis	2022	2255.6 ± 990 ^a^	5971.7 ± 1253 ^b^	5942 ± 1495 ^b,c^	5366 ± 794 ^b^	11,726.7 ± 5669 ^a.c^	**14,118.5** ± 6335 ^a^	7512.2 ± 1739 ^a,b^
	2023	2425.1 ± 718 ^b^	6532.8 ± 991 ^d^	7297.5 ± 1499 ^a,c,d^	5487.9 ± 1862 ^c^	14,128.7 ± 5295 ^a^	**14,202.8** ± 3796 ^a^	7930.1 ± 1357 ^a,c,d^
	2022 ↔ 2023	NS	NS	* *p* = 0.047	NS	NS	NS	NS

Mean ± SD, n: 18–24. ^a,b,c,d^ indicate the significantly different groups (*p* < 0.005). Data of upper and lower epidermis were analyzed separately. (One-way ANOVA, analysis of variance, on ranks, then multiple comparison by Dunn’s method.) * shows the significant differences between the same parameters in 2022 and in 2023 (NS, not significant, because *p* > 0.05); these data were compared by paired *t*-test. The biggest values of categories are highlighted in bold.

**Table 3:** Numbers of stomata measured in 2022 and 2023.

Pc/mm^2^		*Convallaria majalis*	*Allium ursinum*	*Colchicum autumnale*
upper epidermis	2022	266.67 ± 70.08 ^b^	0 ^c^	488.00 ± 41.31 ^a^
	2023	314.29 ± 53.81 ^b^	0 ^c^	460.00 ± 52.57 ^a^
lower epidermis	2022	486.67 ± 127.54 ^a^	430.00 ± 18.52 ^a^	280.00 ± 116.237 ^b^
	2023	400.00 ± 74.07 ^b^	500.00 ± 64.140 ^a*^	340.00 ± 79.09 ^b^
	2022 ↔ 2023	NS	* *p* = 0.005	NS

Mean ± SD, n: 15–22. ^a,b,c^ indicate the significantly different groups (*p* < 0.005). Data from the upper and lower epidermis were analyzed separately. (One-way ANOVA, analysis of variance, on ranks, then multiple comparison by Holm–Sidak method, or Mann–Whitney Rank Sum *t*-test for comparison of the upper and lower epidermises of *Convallaria* to those of *Colchicum*). Between data of 2022 and 2023, there were statistically significant differences only for *Allium* according to the paired *t*-test (Mann–Whitney Rank Sum *t*-test). * shows the significant differences between the same parameters in 2022 and in 2023 (NS, not significant, because *p* > 0.05).

## Data Availability

The data presented in this study are available upon request from the corresponding author.

## Supplementary Materials

The following supporting information can be downloaded at https://www.mdpi.com/article/10.3390/plants14152377/s1. Table S1: Some cases of poisonings caused by misidentification of *Allium ursinum* leaves. (The table includes cases of poisoning where people consumed leaves of *Convallaria majalis* or *Colchicum autumnale* instead of the edible leaves of *Allium ursinum*.) Figure S1: TS of *Convallaria majalis* leaf at the midvein supported with sclerenchyma fibers of varying degrees of lignification and non-lignified collenchyma tissue. Figure S2: Impressions of both upper and lower surfaces of *Convallaria majalis*, *Allium ursinum* and *Colchicum autumnale* fresh leaves prepared with clear nail polish. Figure S3: Impressions of both upper and lower surfaces of *Convallaria majalis* dried leaves prepared with clear nail polish. Figure S4: Impressions of both upper and lower surfaces of *Allium ursinum* dried leaves prepared with clear nail polish. Figure S5: Impressions of both upper and lower surfaces of *Colchicum autumnale* dried leaves prepared with clear nail polish. Figure S6. Adaxial epidermis of *Colchicum autumnale*. (The paralell longitudinal veins are connected with transversal veins and below the epidermis cells the rays of palisade cells of mesophyll are visible). Figure S7: TS of *Allium ursinum* leaf with the subepidermal laticifer cells.
