# Supplementary material for: Histological Features Detected for Separation of the Edible Leaves of Allium ursinum L. from the Poisonous Leaves of Convallaria majalis L. and Colchicum autumnale L
Source: Plants (Basel). 2025 Aug 1;14(15):2377. doi: 10.3390/plants14152377 (PMC12348878; doi:10.3390/plants14152377)
Supplement: Supplementary file 1 [file plants-14-02377-s001.zip › plants-3709414_ FigsS1_S7_M-Hamvas et al 2025.pdf]

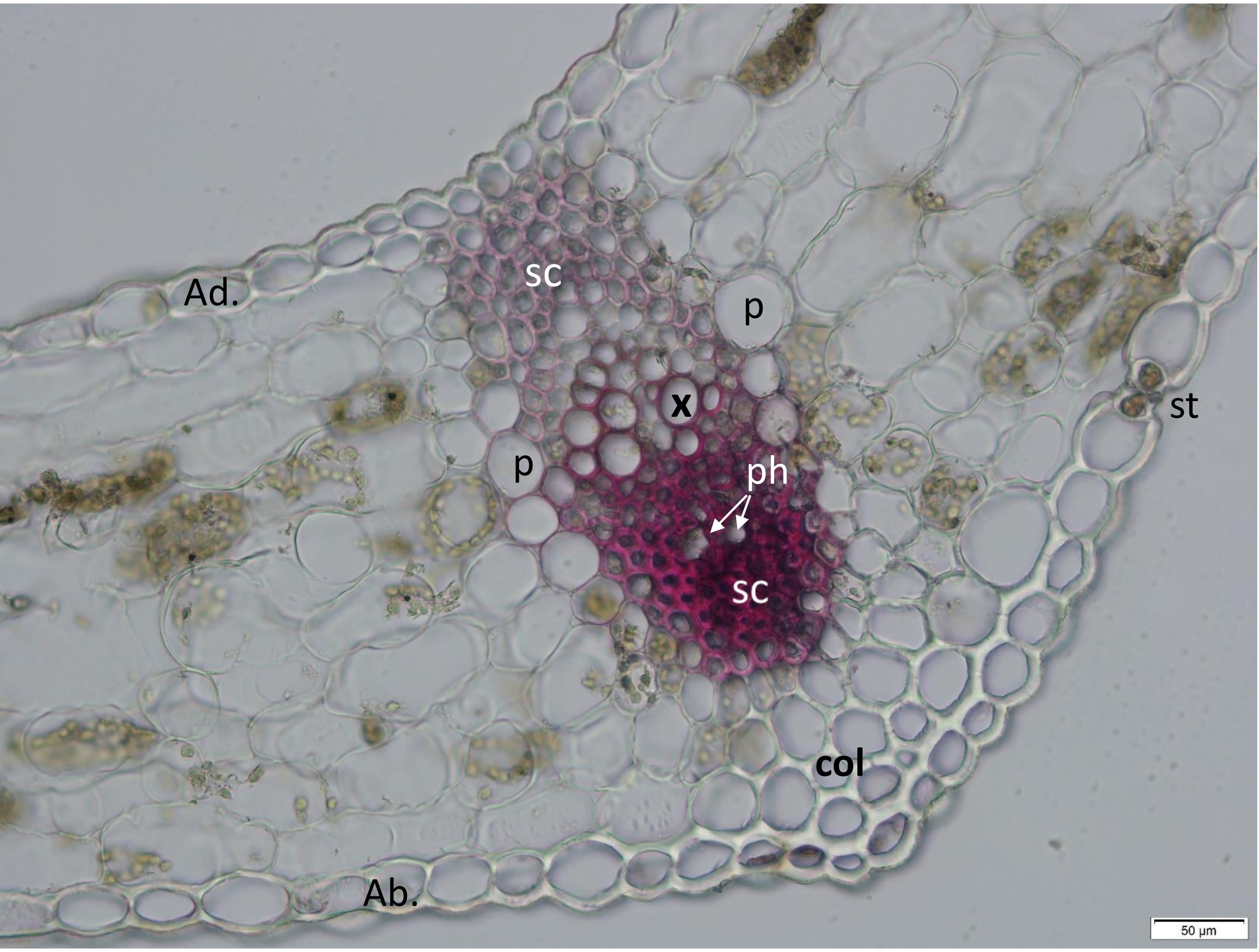

Supplementary Figure S1. TS of *Convallaria majalis* leaf at the midvein supported with sclerenchyma fibres of varying degrees of lignification and non-lignified collenchyma tissue (Ad.: adaxial, upper epidermis; Ab.: abaxial, lower epidermis; col: collenchyma; sc: sclerenchyma fibres; x: xylem; ph: phloem elements; p: parenchymatic sheath; st: stomata). Preparation is stained with Phloroglucinol-HCl, the intensity of red color shows the degree of cell-wall lignification. Bar: 50 µm.

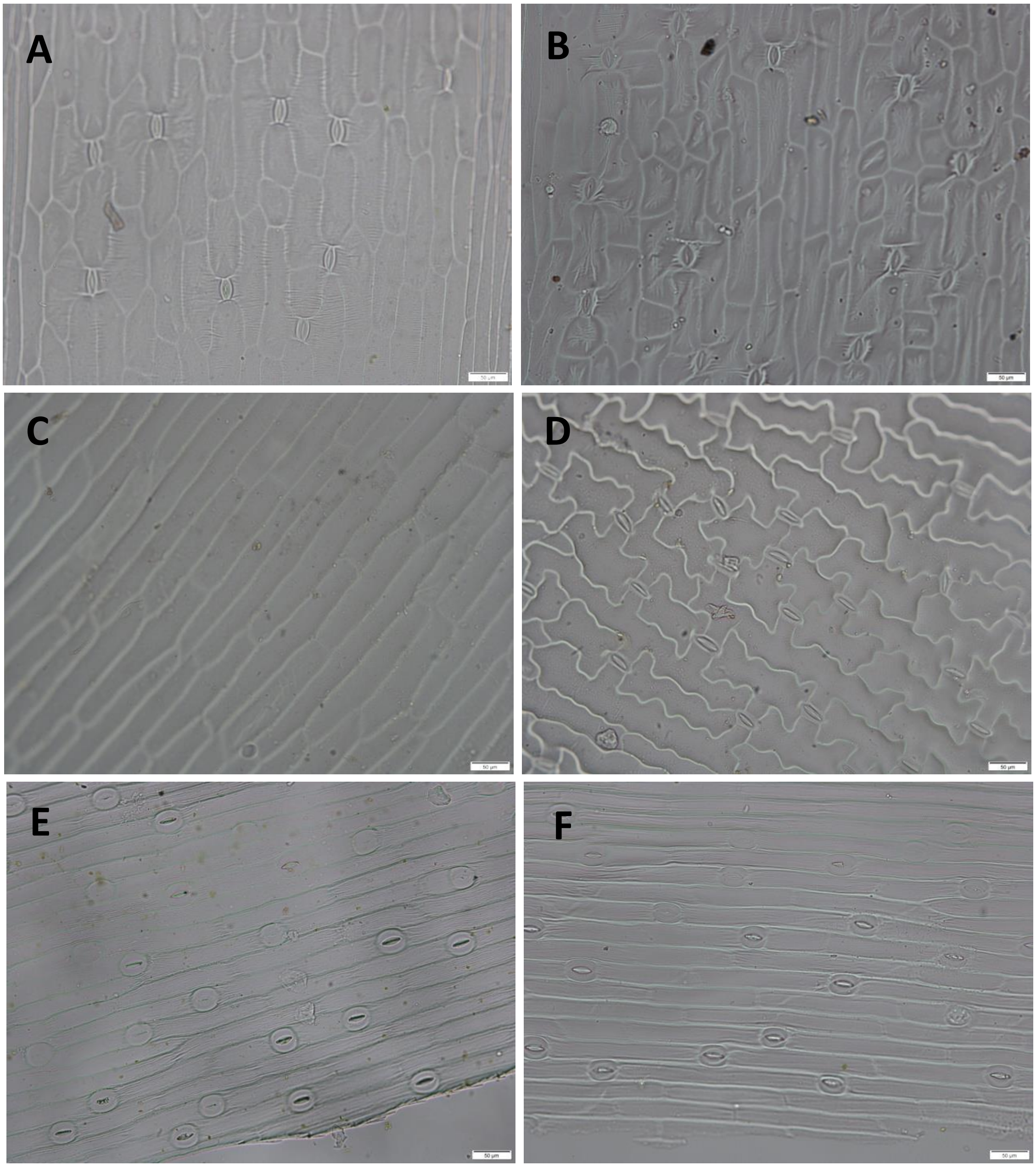

Supplementary Figure S2. Impressions of both, - upper and lower – surfaces of *Convallaria majalis*, *Allium ursinum* and *Colchicum autumnale* fresh leaves prepared with clear nail polish. *Convallaria majalis* (A-B), *Allium ursinum* (C-D) and *Colchicum autumnale* (E-F). Adaxial surfaces: A,C, and E, abaxial surfaces: B,D and F. Bars: 50 μm

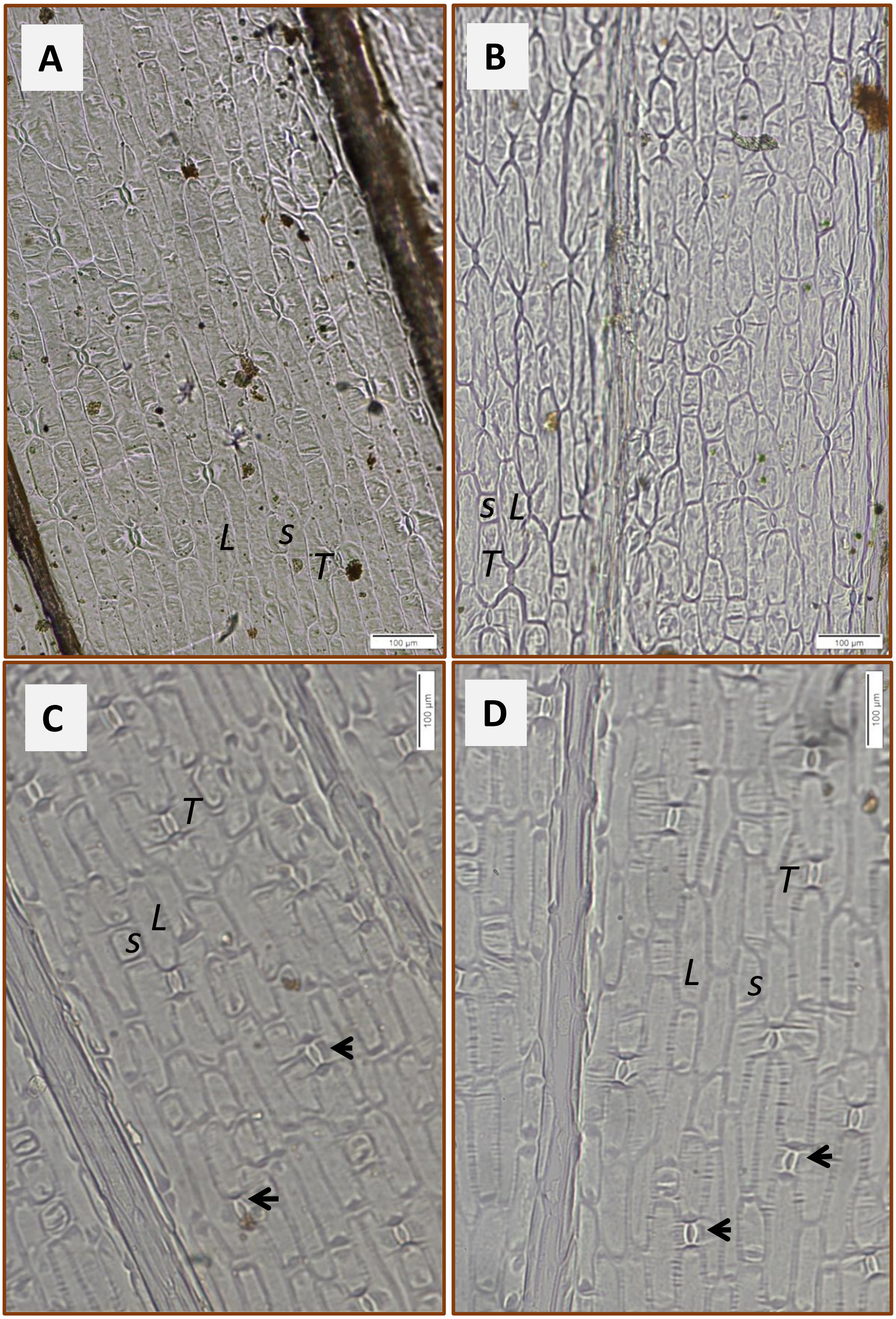

Supplementary Figure S3. Impressions of both, - upper and lower – surfaces of *Convallaria majalis* dried leaves prepared with clear nail polish. Adaxial surfaces: A and C, abaxial surfaces: B and D. Leaves are pressed and dried, because of beeing specimens in „Herbarium" Collection of plants in University of Debrecen, Hungary. Plants were collected in different regions of Hungary and at different times; collected in Tokaj Montains, Hungary (A-B) and in Keszthelyi Montains, Hungary (C-D). Bars: 100 µm. Among pavement cells the short (*S*), long (*L*), *T* cells and stomatal-complexes (arrows) are well distinguishable.

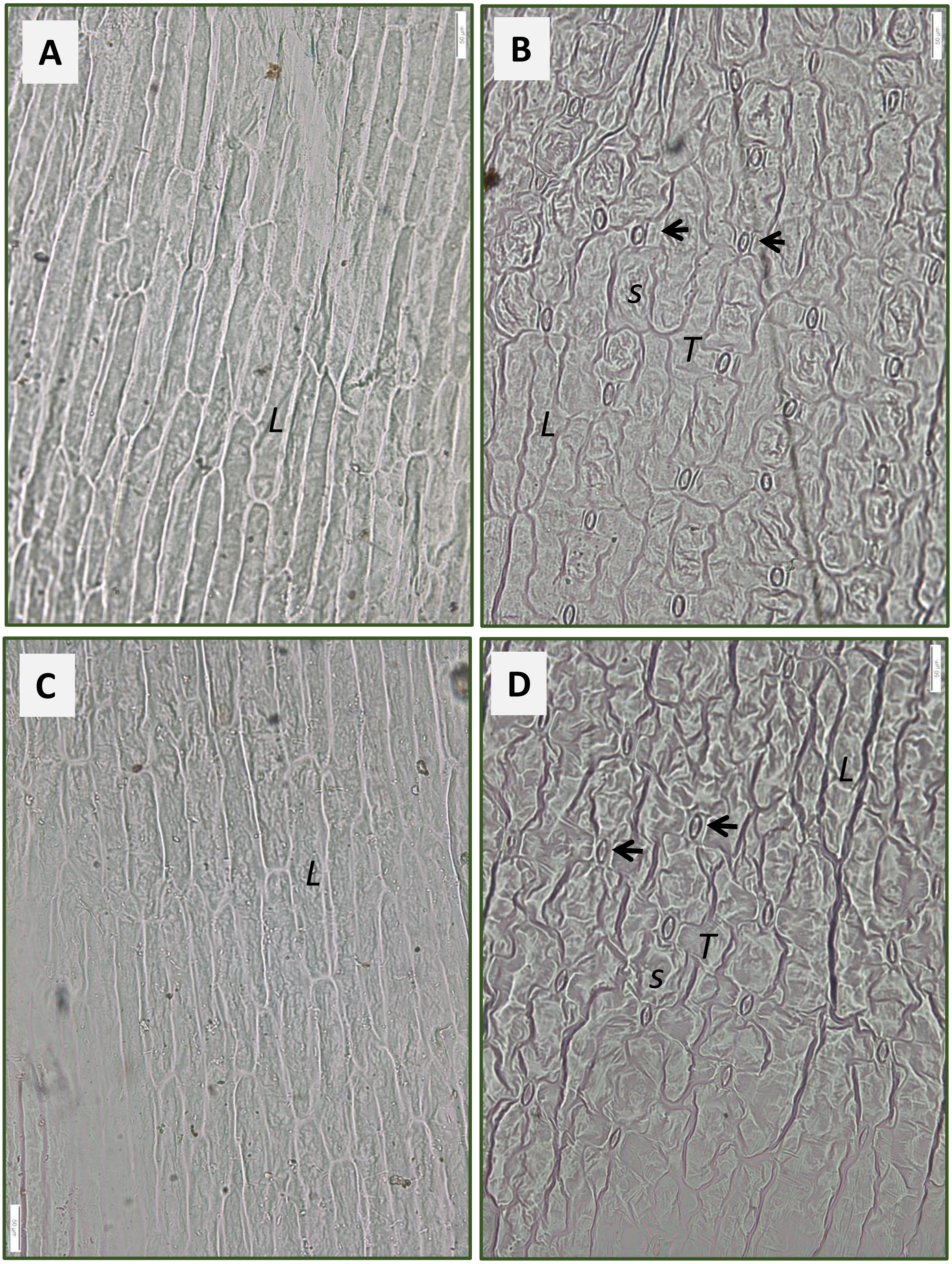

Supplementary Figure S4. Impressions of both, - upper and lower – surfaces of *Allium ursinum* dried leaves prepared with clear nail polish. Adaxial surfaces: A and C, abaxial surfaces: B and D. Leaves are pressed and dried, because of beeing specimens in „Herbarium" Collection of plants in University of Debrecen, Hungary. Plants were collected in different regions of Europe and at different times: in the Keszthelyi Montains, Hungary (A-B) and in South Romania (C-D). Bars: 50 μm. On lower preparations among pavement cells the short (*S*), long (*L*), *T* cells and stomata (arrows) are distinguishable.

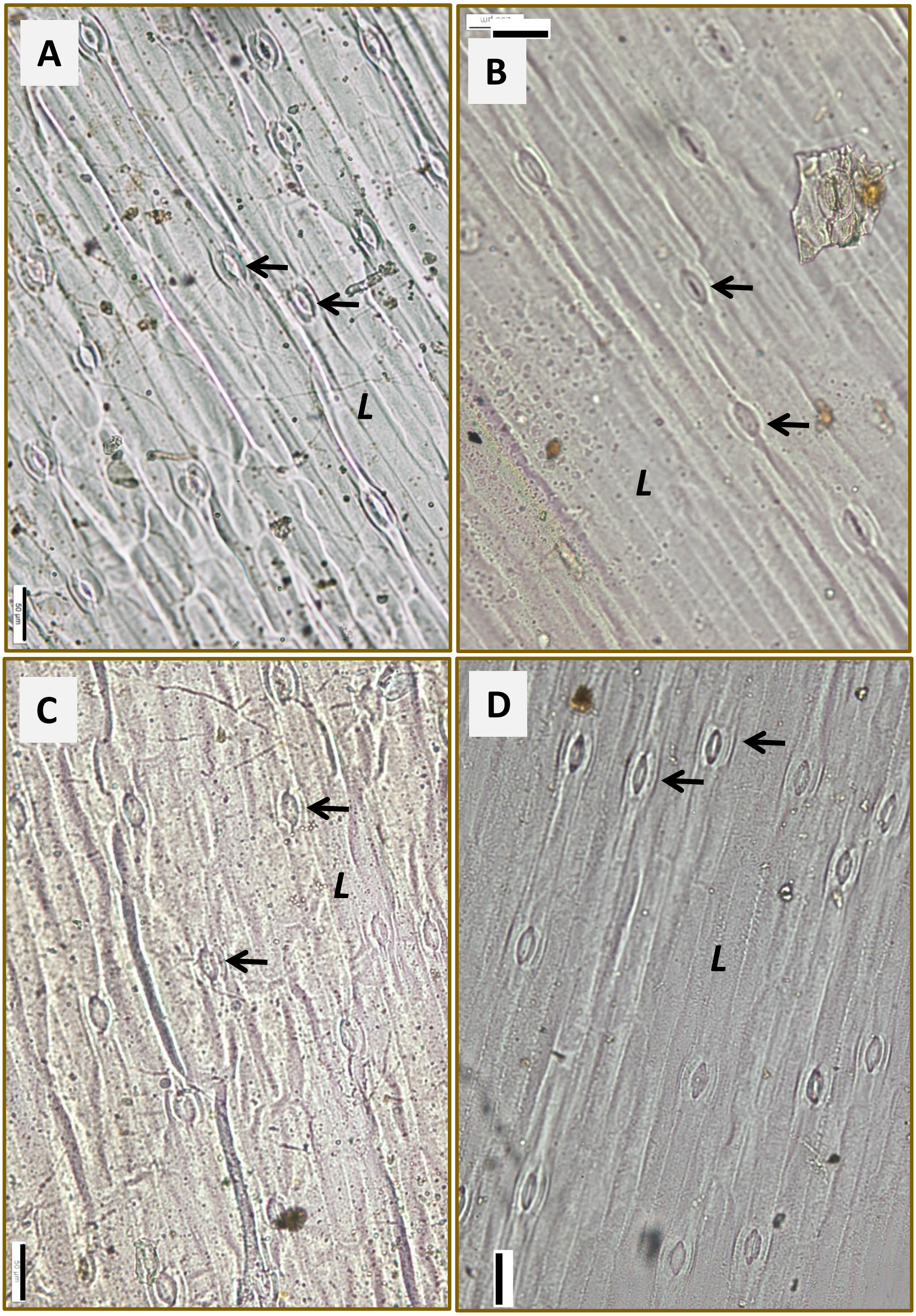

Supplementary Figure S5. Impressions of both, - upper and lower – surfaces of *Colchicum autumnale* dried leaves prepared with clear nail polish. Adaxial surfaces: A and C, abaxial surfaces: B and D. Leaves are pressed and dried, because of beeing specimens in „Herbarium" Collection of plants in University of Debrecen, Hungary. Plants were collected in different regions of Europe and at different times; collected in Slovenia (A-B) and in Kömörő, Hungary (C-D). Bars: 50 µm. Among the elongated (long, *L*) pavement cells there are stomatal-complexes (arrows) on both surfaces.

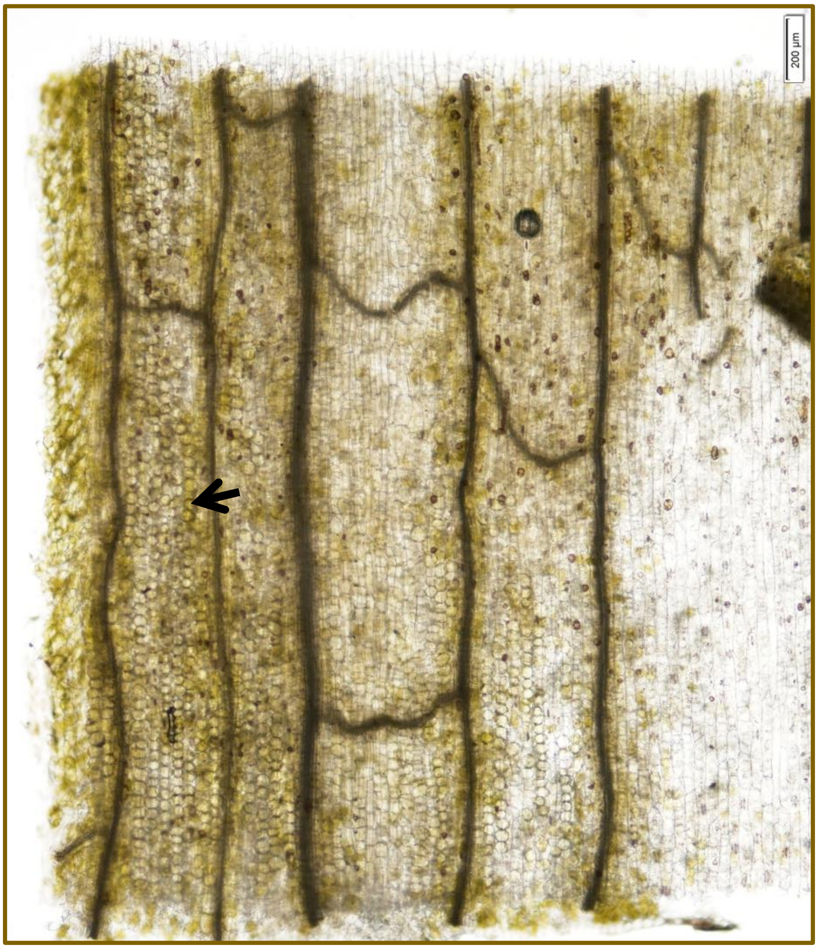

Supplementary Figure S6. Adaxial epidermis of *Colchicum autumnale.* The paralell longitudinal veins are connected with transversal veins. Below the epidermis cells the rays of palisade cells of mesophyll are visible (black arrow). Bar: 200 µm.

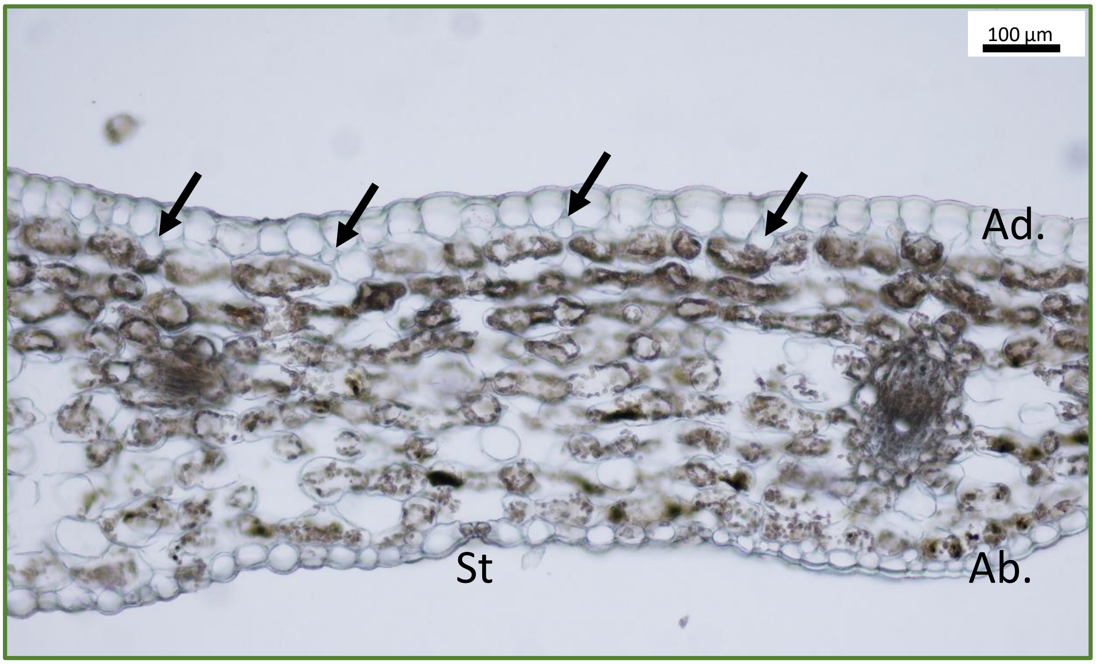

Supplementary Figure S7. TS of *Allium ursinum* leaf with the subepidermal laticifer cells (black arrows, Ad.: adaxial, upper epidermis; Ab.: abaxial, lower epidermis; St: stomatal-complex). Bar: 100 µm.
