# Supplementary material for: Histological Features Detected for Separation of the Edible Leaves of Allium ursinum L. from the Poisonous Leaves of Convallaria majalis L. and Colchicum autumnale L
Source: Plants (Basel). 2025 Aug 1;14(15):2377. doi: 10.3390/plants14152377 (PMC12348878; doi:10.3390/plants14152377)
Supplement: Supplementary file 1 [file plants-14-02377-s001.zip › plants-3709414_Table S1 .pdf]

Table S**1.** Some cases of poisonings caused by misidentification of *Allium ursinum* leaves.
Table does not include suicide and overdose cases. (DE: deceased, Col.: colchicine, SGOT: Serum glutamic oxaloacetic transaminase, CK: creatinine kinase, LDH: Lactate dehydrogenase, P.I.C.: Poisons Information Centre, P.C.C: Poison Control Center or P.C. : Poison Control Center, n.p.: not published in the article)

**Instead of *Allium ursinum* consumed plant species**	**number of patients** (country, date)	**symptons**	**outcome**	**some data of health care observations (HO) / post mortem observations (PMO) or / and laboratory analysis (LA)**	**reference**
Convallaria majalis	29 (1995-2007, Italy)	yes, but not published	cases were evaluated as, "the concentration of cardioactive glycosides in the leaves is comparatively low so the life-threatening conditions due to poisoning seldom occure"	n.p.	data of Milan P.C. Niguarda Hospital, published by Colombo et al., 2009. [33]
	one person in the north-west of Italy, (Piedmont region, 2008-2009)	yes (but not published)	n. p.	n.p.	registered by the Milan P.C.C. at Niguarda Hospital, published by

					Davanzo et al., 2011. [35]
	87-year-old woman suffering from advanced dementia	digestive disorders, blood pressure of 155/85 mm Hg, a heart rate of 45 bpm, ECG showed sinus bradycardia at 45 bpm and repolarization disorders in the anterior and lateral derivations.	**Recover -** after 24h intensive health care with continuous hemodynamic and serum digoxin and K^+ concent-ration monitoring, -simple symptomatic treatment-.	LA: Her kalemia was 4.4 mmol/L, plasma creatinine was 66 µmol/L, and troponin was <0.06 µg/L. Her digoxin serum concentration was in the therapeutic range.	Alexandre et al., 2012. [48]
	89 (there is no data on those who have been proven to have eaten leaves!) (Norway, 2008)	„The most common situations concern small children ingesting a small number of the red berries, present in the late summer and autumn.	cases were evaluated as, "because of the limited amount ingested, such intakes are usually benign."	In average it can cause: cardiotoxic effects like ECG- disturbances and arrhythmias	data of Norwegian P.I.C. published by Spillum and Muan, 2010. [47]
	1272 accidental by consumed leaves of total 1282 (168 adults, 1108 children), (Jan. 1995 – Dec. 2009)	n.p.	n.p.	n.p.	data of Swiss Toxicological Information Centre (STIC), published by Fuchs et al., 2011. [36]

	two children aged 0.5-5 years (1997-2013)	n.p.	It is likely that they recovered, no tragic outcome mentioned.	n.p.	data of Berlin and Freiburg P.I.C. published by Hermanns-Clausen et al., 2019. [38]
Colchicum autumnale	11 (1995-2007, Italy)	profuse vomiting and diarrhea, which can be bloody	2 of 11 DE!	-hypovolemic shock and multisystem organ failure within 24-72 hours, -Subsequent complications include bone marrow suppression with resultant leukopenia, thrombocytopenia, and possibly sepsis. -Coma, convulsions and sudden death occur.	data of Milan P.C. Niguarda Hospital, published by Colombo et al., 2009. [39]
	1		recover		Michael et al., 1999 in Akram et al., 2012. [55]
	1		DE!		
	1	yes (not published)	n.p.	n.p.	registered by the Milan P.C.C. Italy, published by Davanzo et al. (2011) [35]
	20 (2000-2013)		2 DE! 18 recover	n.p.	registered by the P.C.C. of the University Medical Centre in Ljubljana (Slovenia) published by

					Vončina et al., 2014. [45]
	the wife of a couple	she tasted only, she suffered only mild poisoning	recover	n.p.	Sundov et al., 2005. [58]
	the 62-year-old husband of the couple (Croatia)	symptoms of gastroenteritis, then a multi-organ system failure developed	DE! - in hospital	PMO: hepatic centrilobular – and nephrotoxic acute tubular necrosis, petechial bleeding in fatty tissue, blunt and shortened intestinal villi and cerebral toxic edema.<br>LA: highly elevated blood concentrations of hepatic enzymes, creatine kinase, lactate dehydrogenase and blood urea nitrogen, leukocytopenia, thrombocytopenia	
	65-year-old woman (Germany)	sickness, stomach aches and diarrhoea, than comatose	DE! - in hospital	PMO: liver showed diffuse vacuolization in the cytoplasma of hepatocytes, possibly due to the relatively short survival time, only isolated mitotic structures were found within the epithelium of the colon.<br>LA: 65 μg/l Col. in blood and all tested organs contained in equally high levels.	Wollersen et al., 2009. [38]
	66-year –old man (Germany)	typical gastrointestinal symptoms	recover	LA: the elevated activities of SGOT, CK, and LDH were indications of a certain extent of cell damage. 19 μg/L Col. in blood plasma	Peters et al., 2004. [39]
	50-year-old male	weakness, abdominal pain, nausea, vomiting	DE! - in hospital	PMO: brain oedema, lung oedema and congestion, heart weighing 700 g with	Danilović et al., 2020.

	(near Loznica in Serbia)	and diarrhea without blood, then gastroenterocolitis, acute renal faliure, hepatic lesions and cardiorespiratory insufficiency		ventricular hypertrophy, myocardial fibrosis, liver congestion and steatosis, spleen congestion, pancreatic fibrosis, etc., tissue necrosis in liver and kidney were detected<br>LA: Col. in the blood: 0.011 mg/L, it was detected in other organs as well	[34]
	71-year-old woman (Ljubljana, Slovenia)	gastroenterocolitis followed by multi-organ dysfunction, then alopecia	recover	LA: Toxicology analysis revealed: Col. in the patient's gastric lavage, blood (5 μg/l) and urine (30 μg/l).	Brvar et al., 2004.<br>[67]
	the 60-year-old wife (with controlled hypertonia) of a couple	vomiting, diarrhaea, abdominal discomfort	recover (after 2 weeks)	ECG: sinus rhythm with right bundle branch block. Abdominal ultrasound: uneven, increased liver echogenicity, and cortical widening and decreased echogenicity of the kidneys. LA: list of the data are published	Brncic et al., 2001.<br>[40]
	the 64-year-old husband of the couple (Mrkopalj, in Gorski Kotar, Croatia)	severe form of gastrointestinal irritation	DE	HO: In the 16th hour irreversible cardiovascular collapse occurred. (LA: data are published, PMO: presence of Col. and related alkaloids in the remnants)	
	a 16-year-old boy	nausea, vomiting and watery diarrhea, colapse	recover after intensive heath care		Klintschar et al., 1999.<br>[41]
	a 44 year-old man (England?)	multi-organ derangement	DE - in hospital	PMO: hemorrhagic lung oedema, hypocellular bonemarrow, centrilobular fatty necrosis of the liver,	

				necrosis of the proximal convoluted tubuli of the kidneys<br>LA: 7.5 μg/ml Col. in the bile but no substance was detected in the postmortem blood	
	an old couple (Germany?)		both DE at home	Toxicological analyses (LC-MS/MS): Col. 36.6 - 98.3 ng/mL in the heart blood and 22.7 - 78.4 ng/mL in the femoral blood of the victims. PMO - histology: advanced autolysis, etc.	Wehner et al., 2006.<br>[68]
	43-yr-old female	severe multiorgan injury	recover after 3 weeks intensive health care therapy (treatments: supportive care, antibiotic therapy, and granulocyte-directed growth factor)	3 years after recovery the patient continued to complain of muscle weakness and intermittent episodes of hair loss.	Gabrscek et al., 2004.<br>[42]
	a group in Piedmont region, Italy	no symptons	???no intoxications	treatments in hospital	Davanzo et al., 2011. [35]
	212 of the total 231 Col. poisonings (127 adults, 99 children)<br>Jan. 1995 – Dec. 2009		3 cases were fatal (DE):<br>1. a 3-year-old boy<br>2. a 57-old<br>3. a 62-year-old patient	1. Bradycardia, cerebral edema, vomiting, seizures, respiratory and hepatic failure, Col. concentration in serum: 7 μg/l<br>2. Pulmonary edema, arrhythmia, coagulopathy, renal failure<br>3. Renal failure, necrosis of the heart muscle, coagulopathy	data of STIC published by Fuchs et al., 2011.<br>[36]

	4 children aged 0.5-5 years (out of 42.344 confirmed exposures to 227 plant species, during 1997-2013)	the consumed leaves caused furry feeling in throat, nausea, vomiting (repeated and persistent), diarrhea	n.p.	Electrolyte disturbances, desiccosis, agitation, multi-organ failure	data of two (Berlin and Freiburg) German P.I.C. published by Hermanns-Clausen et al., 2019. [37]
